# Bayesian networks may allow better performance and usability than logistic regression

**DOI:** 10.1186/s13054-024-05015-w

**Published:** 2024-07-11

**Authors:** Jared M. Wohlgemut, Erhan Pisirir, Rebecca S. Stoner, Evangelia Kyrimi, Barbaros Yet, William Marsh, Zane B. Perkins, Nigel R. M. Tai

**Affiliations:** 1 Centre for Trauma Sciences, Blizard Institute, Queen Mary University of London, 4 Newark Street, London, E1 2AT UK; 2 Machine Intelligence and Decision Support (MInDS) Research Group, School of Electronic Engineering and Computer Science, Digital Environment Research Institute, Queen Mary University of London, London, UK; 3 Department of Cognitive Science, Graduate School of Informatics, Middle East Technical University, Ankara, Turkey; 4 Royal London Hospital, Barts NHS Health Trust, London, UK; 5 London’s Air Ambulance, London, UK; 6 Royal Centre for Defence Medicine, Birmingham, UK

We read with great interest the article by Brac et al. entitled “Development and validation of the TIC score for early detection of traumatic coagulopathy upon hospital admission: a cohort study” [1]. We congratulate the authors on their work focusing on trauma-induced coagulopathy (TIC), a key outcome early after trauma that increases the risk of mortality and may be treated and potentially reversed if promptly identified [2]. The study demonstrates a simple screening tool for early detection of TIC (defined as PTr > 1.2). The tool was developed using a multivariate regression analysis where coefficients were translated into more easy-to-use integers derived from binary variables. These variables at admission to a trauma center were: point-of-care haemoglobin < 11 g/dL, shock index > 0.9, Glasgow Coma Scale < 9, prehospital fluid resuscitation > 1000 ml, and prehospital norepinephrine. The score achieved an area under the receiver operator curve (AUROC) of 0.82 in the training dataset (n = 984), 0.80 in the validation dataset (n = 2275), 0.93 in the prospective dataset (n = 230), and 0.83 overall (n = 3489).

The authors commented that our previously-developed Bayesian Network (BN) score [3], which also predicts PTr > 1.2, had similar performance but is “not suitable for the early management of severely injured patients because of its complexity (14 variables including 3 laboratory variables) that precludes its timely calculation at the admission to the trauma center”. We respectfully refute this assertion: we designed the tool precisely for use in the early phase of trauma resuscitation.

Firstly, we recognised that the complex set of inter-dependent physiological and injury variables that determine the development of TIC merit a sophisticated approach to modelling. Compared to logistic regression models—which apply fixed coefficients to a pre-determined list of variables, all of which must be present to calculate an output—BNs allow the causal modelling of complex systems and enable the incorporation of data from meta-analyses, expert knowledge and data, mitigating the risk of over-fitting and enhancing generalisability [3]. BNs can account for non-linear and hierarchical relationships between multiple continuous and categorical variables in data. Contrastingly, in regression models such as that employed by Brac et al. [4] continuous data are dichotomised, which reduces precision, especially in the case of non-linear relationships between predictors and the outcome, and does not exploit the richness of the data. These design choices enabled excellent overall performance of our BN model, measured by discrimination (AUROC 0.93 versus 0.83 compared to Brac et al.) and calibration (Brier score 0.06 versus 0.115). It stands to reason that a BN is more “suitable for the early management of severely injured patients” than a logistic regression model if the BN is better at predicting the desired outcome (TIC).

Secondly, we recognised that there is considerable uncertainty in early trauma [5]. Prediction tools should acknowledge this by permitting prediction even in the absence of some modelled variables. The statistical strength of the conditional probabilities employed in our model is robust enough to withstand absent variables, which are calculated using the prior probabilities. This permits updating predictions as more information becomes available and precision increases with more information. In a limited prospective evaluation, AUROC was 0.77 within seconds of arrival to the Resuscitation Bay of our Emergency Department, 0.84 within 3 min, and 0.87 within 6–15 min (with results from point-of-care arterial blood gas analysis) [6]. In other words, a prediction could be calculated sooner (with incomplete data) than a 5-input logistic regression, and if the decision can wait a few minutes, our BN model delivers a more accurate result. In contrast, logistical regression models cannot work with missing variables.

Thirdly, we recognised that whether a risk prediction is used depends on much more than simply providing information in a timely manner. Factors that may affect the adoption of a decision-support system in pre-hospital or hospital trauma care include its predictive accuracy, trustworthiness, usability, usefulness, understandability, and availability [7]. The best model for an end user may not necessarily be the simplest model. With modern computing power and user interface/user experience (UI/UX) design, there may no longer be a need to sacrifice model performance to achieve usability.

## Data Availability

Not applicable.

## Abbreviations

TIC: Trauma-induced coagulopathy
AUROC: Area under the receiver operator curve
BN: Bayesian network
UI/UX: User interface/user experience

## References

[1] Brac L, Levrat A, Vacheron C-H, Bouzat P, Delory T, David J-S. Development and validation of the tic score for early detection of traumatic coagulopathy upon hospital admission: a cohort study. Crit Care. 2024;28(1):168. 10.1186/s13054-024-04955-7. 38762746 10.1186/s13054-024-04955-7 PMC11102139

[2] Moore EE, Moore HB, Kornblith LZ, Neal MD, Hoffman M, Mutch NJ, Schöchl H, Hunt BJ, Sauaia A. Trauma-induced coagulopathy. Nat Rev Dis Primers. 2021. 10.1038/s41572-021-00264-3. 33927200 10.1038/s41572-021-00264-3 PMC9107773

[3] Yet B, Perkins Z, Fenton N, Tai N, Marsh W. Not just data: a method for improving prediction with knowledge. J Biomed Inform. 2014;48:28–37. 10.1016/j.jbi.2013.10.012. 24189161 10.1016/j.jbi.2013.10.012

[4] Royston P, Altman DG, Sauerbrei W. Dichotomizing continuous predictors in multiple regression: a bad idea. Stat Med. 2006;25(1):127–41. 10.1002/sim.2331. 16217841 10.1002/sim.2331

[5] Wohlgemut JM, Marsden MER, Stoner RS, et al. Diagnostic accuracy of clinical examination to identify life- and limb-threatening injuries in trauma patients. Scand J Trauma Resuscit Emerg Med. 2023;31(1):18. 10.1186/s13049-023-01083-z. 10.1186/s13049-023-01083-z PMC10082501 37029436

[6] Mossadegh S. Application and development of bayesian networks for predictive modelling of coagulopathy and mortality in trauma patients. Queen Mary University of London; 2019.

[7] Kyrimi E, Dube K, Fenton N, et al. Bayesian networks in healthcare: what is preventing their adoption? Artif Intell Med. 2021;116:102079. 10.1016/j.artmed.2021.102079. 34020755 10.1016/j.artmed.2021.102079

